# Effects of simvastatin on white matter integrity in healthy middle‐aged adults

**DOI:** 10.1002/acn3.51421

**Published:** 2021-07-18

**Authors:** Nicholas M. Vogt, Jack F. V. Hunt, Yue Ma, Carol A. Van Hulle, Nagesh Adluru, Richard J. Chappell, Karen K. Lazar, Laura E Jacobson, Benjamin P. Austin, Sanjay Asthana, Sterling C. Johnson, Barbara B. Bendlin, Cynthia M. Carlsson

**Affiliations:** ^1^ Wisconsin Alzheimer’s Disease Research Center University of Wisconsin School of Medicine and Public Health Madison Wisconsin; ^2^ Waisman Laboratory for Brain Imaging and Behavior Waisman Center University of Wisconsin‐Madison Madison Wisconsin; ^3^ Department of Biostatistics and Medical Informatics University of Wisconsin School of Medicine and Public Health Madison Wisconsin; ^4^ Geriatric Research Education and Clinical Center William S. Middleton Memorial Veterans Hospital Madison Wisconsin; ^5^ Geriatrics Division Department of Medicine University of Wisconsin School of Medicine and Public Health Madison Wisconsin; ^6^ Wisconsin Alzheimer’s Institute University of Wisconsin School of Medicine and Public Health Madison Wisconsin

## Abstract

**Background:**

The brain is the most cholesterol‐rich organ and myelin contains 70% of total brain cholesterol. Statins are potent cholesterol‐lowing medications used by millions of adults for prevention of vascular disease, yet the effect of statins on cholesterol‐rich brain white matter (WM) is largely unknown.

**Methods:**

We used longitudinal neuroimaging data acquired from 73 healthy, cognitively unimpaired, statin‐naïve, middle‐aged adults during an 18‐month randomized controlled trial of simvastatin 40 mg daily (*n* = 35) or matching placebo (*n* = 38). ANCOVA models (covariates: age, sex, *APOE*‐ɛ4) tested the effect of treatment group on percent change in WM, gray matter (GM), and WM hyperintensity (WMH) neuroimaging measures at each study visit. Mediation analysis tested the indirect effects of simvastatin on WM microstructure through change in serum total cholesterol levels.

**Results:**

At 18 months, the simvastatin group showed a significant preservation in global WM fractional anisotropy (β = 0.88%, 95% CI 0.27 to 1.50, *P* = 0.005), radial diffusivity (β* = *−1.10%, 95% CI −2.13 to −0.06, *P* = 0.039), and WM volume (β* = *0.72%, 95% CI 0.13 to 1.32, *P* = 0.018) relative to the placebo group. There was no significant effect of simvastatin on GM or WMH volume. Change in serum total cholesterol mediated approximately 30% of the effect of simvastatin on WM microstructure.

**Conclusions:**

Simvastatin treatment in healthy, middle‐aged adults resulted in preserved WM microstructure and volume at 18 months. The partial mediation by serum cholesterol reduction suggests both peripheral and central mechanisms. Future studies are needed to determine whether these effects persist and translate to cognitive outcomes.

**Trial Registration:**

NCT00939822 (ClinicalTrials.gov).

## Introduction

The brain is the most cholesterol‐rich organ and contains nearly a quarter of total body cholesterol.^1^ Within the brain, cholesterol exists in two primary pools—approximately 70% of brain cholesterol is found in myelin sheaths and the remainder is located in the cell membranes of neurons and astrocytes.^2^ Cholesterol is required for myelination^3^ and contributes to the insulating properties of mature myelin by reducing ion permeability.^1^ Cholesterol homeostasis in the brain is independent of peripheral cholesterol metabolism and is maintained by complex regulatory mechanisms. Because the blood–brain barrier (BBB) prevents uptake of peripheral cholesterol from circulating lipoproteins, essentially all brain cholesterol is synthesized *de novo*,^2^ with significantly greater production in glia than in neurons.^4^ Additionally, while cholesterol in peripheral tissues undergoes turnover every few days, brain cholesterol has an extremely long half‐life of 6 months to 5 years^5^ due to highly efficient recycling processes.

Statins are potent cholesterol‐lowering medications used by millions of adults for the treatment of hypercholesterolemia and prevention of cardiovascular and cerebrovascular disease. By inhibiting HMG‐CoA reductase (the rate‐limiting enzyme in cholesterol synthesis), statins decrease cholesterol production and increase clearance of low‐density lipoprotein (LDL) particles, which results in a reduction in peripheral circulating cholesterol levels.^6^ Several statins (including simvastatin) can cross the BBB to enter the central nervous system (CNS), where they not only inhibit brain cholesterol synthesis, but also have pleotropic effects.^7^ Despite the widespread use of statins, it is largely unknown how these medications affect cholesterol‐rich white matter (WM) brain regions in humans.

Diffusion tensor imaging (DTI) is an in vivo neuroimaging technique that has been widely used to study WM microstructure.^8^ By measuring the diffusion of water molecules within tissues, DTI provides quantitative information regarding alterations in WM integrity (e.g., axonal and myelin damage). A limited number of previous studies have attempted to assess the influence of statins on WM microstructure using DTI.^9^, ^10^ However, these studies have been observational in nature, and are potentially confounded by the statin‐exposed individuals having significantly elevated cardiovascular disease risk factors, which can also affect WM microstructure. Thus, causative effects of statins on WM microstructure remain to be determined.

In this study, we took advantage of longitudinal neuroimaging data acquired during an 18‐month randomized controlled trial of simvastatin in healthy, statin‐naïve, cognitively unimpaired, middle‐aged adults. We tested the effect of simvastatin treatment on longitudinal global and regional WM microstructure (as measured by DTI metrics), as well as longitudinal WM volume (derived from conventional T1‐weighted MRI), and white matter hyperintensity (WMH) lesion volume. Additionally, to determine if the effects of simvastatin were specific to WM, we assessed the effect of simvastatin on longitudinal gray matter (GM) volume. Finally, in order to investigate potential causal mechanisms, we tested whether the effect of simvastatin on WM microstructure was mediated by changes in peripheral cholesterol levels.

## Methods

### Study design and participants

This secondary analysis used data acquired from a prospective, double‐blind, randomized controlled trial designed to evaluate the effects of simvastatin on beta amyloid pathology as indexed by CSF biomarkers in healthy, cognitively unimpaired, middle‐aged (40–72 years old) adults with a parental family history of Alzheimer’s disease dementia (ClinicalTrial.gov identifier: NCT00939822).^11^ Main exclusion criteria included current use of cholesterol‐lowering medications, contraindication to use of statins, or history of known cardiovascular disease requiring use of statin (see Table 1 for full eligibility criteria). In the original trial, there was no effect of simvastatin over 18 months on the primary endpoint of changes in CSF beta amyloid or secondary endpoints of changes in CSF soluble amyloid precursor protein or tau protein.

**Table 1:** Full eligibility criteria for study.

Inclusion criteria
Parental history of probable or definite Alzheimer’s disease dementia
Age 40–72 years
Exclusion criteria
Dementia or mild cognitive impairment (MCI) on screening evaluation
Current use of cholesterol‐lowering medication
History of liver disease (cirrhosis, hepatitis, or elevation of AST or ALT > 2 times upper limits of normal)
History of adverse reaction to statin drug
Elevated CK (>2 times upper limit of normal)
Use of medications known to interact with statins, including: erythromycin, clarithromycin, digoxin, itraconazole, ketoconazole, fluconazole, nefazodone, cyclosporine, protease inhibitors, amiodarone, verapamil, warfarin, or investigational drug in another trial
Use of large quantities of grapefruit juice (> 1 quart/day)
Elevated creatinine (> 1.8 mg/dL at baseline)
Pregnancy
Previous history of lumbar spine surgery with contraindication to lumbar puncture Contraindication to lumbar puncture due to use of warfarin or other anticoagulants
Claustrophobia requiring sedation for MRI
Pacemaker or other contraindication to Gd‐enhanced MRI
History of MI, significant arterial occlusive disease, DM, or stroke
LDL‐C > 160 mg/Dl and two or more CHD risk factors

**Figure 1:**
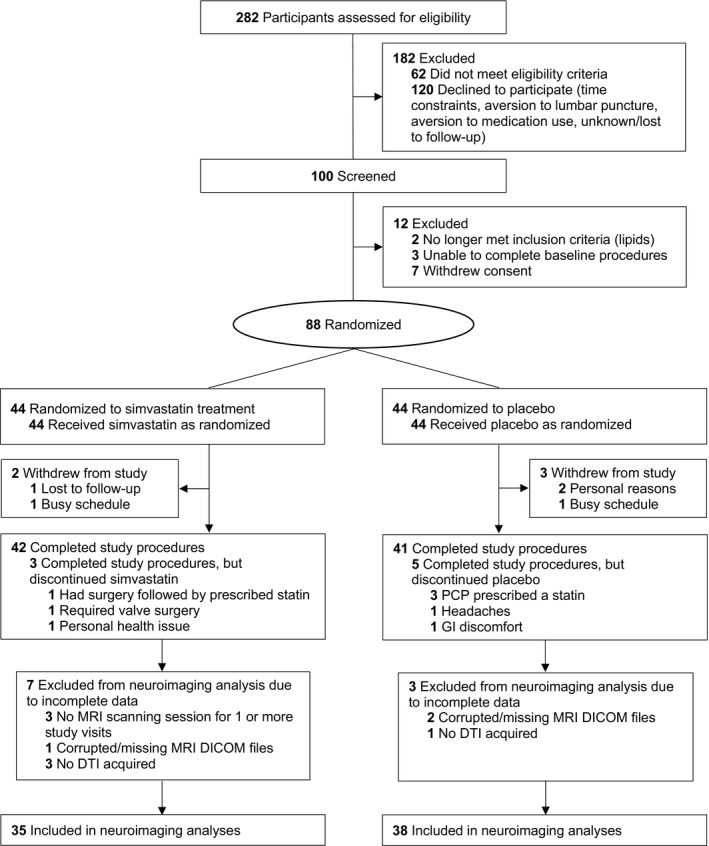
Flow diagram of study trial enrollment, randomization, treatment, and exclusion criteria for neuroimaging analyses.

Eligible participants underwent a screening visit and a total of 88 participants were randomly assigned to receive simvastatin (40 mg, daily) in the treatment group (*n* = 44), or matching placebo in the control group (*n* = 44) for 18 months (see Fig. 1 for flowchart of trial enrollment and Supplemental Table S1 for participant characteristics of full original study population). Participants underwent fasting blood draw and MRI at baseline, 6‐, 12‐, and 18‐month study visits. A total of 73 participants (*n* = 38 placebo, *n* = 35 simvastatin) had complete neuroimaging data and were included in the current analyses. The University of Wisconsin Health Sciences Institutional Review Board approved all study procedures and all participants provided written informed consent. Data were collected from November 24, 2009 to September 17, 2013.

### MRI acquisition details

Diffusion‐weighted images were acquired using a spin‐echo, single‐shot, echo‐planar imaging pulse sequence (8 × *b* = 0 s/mm^2^, 40 × *b* = 1300 s/mm^2^; TR/TE = 8000/84.5 ms; flip angle = 90°; FOV = 240 mm; 96 × 96 acquisition matrix; 2.5 × 2.5 × 2.5 mm^3^ isotropic voxel resolution). T1‐weighted structural images were acquired using a 3D inversion recovery prepared fast spoiled gradient‐echo FSPGR‐BRAVO sequence (TI = 450 ms; TR/TE = 8.1/3.2 ms; flip angle = 12°; FOV = 256 mm; 256 × 256 acquisition matrix; 1 × 1 × 1 mm^3^ isotropic voxel resolution). T2‐weighted images were acquired using a 3D fluid‐attenuated inversion recovery (FLAIR) pulse sequence (TI = 1871 ms; TR/TE = 6000/122.3 ms; flip angle = 90°; FOV = 256 mm; 256 × 256 acquisition matrix; 1 × 1 × 1 mm^3^ isotropic voxel resolution).

### MRI processing

Diffusion‐weighted images were denoised,^12^ corrected for Gibbs ringing,^13^ and corrected for motion and eddy current distortions.^14^ DTI parameter maps (fractional anisotropy [FA], mean diffusivity [MD], radial diffusivity [RD], and axial diffusivity [AD]) were fit using Diffusion Imaging in Python (DIPY)^15^ and visually inspected prior to further processing. Mean DTI measures were extracted using subject‐space total WM masks. Total WM masks were created using the *Atropos* ^16^ tool in ANTs^17^ to segment FA images into WM fraction maps, which were thresholded at 0.7 and binarized. For each time point for each participant, DTI parameter maps were masked using total WM masks and *fslstats* ^18^ was used to extract mean DTI measures in total WM. For additional region of interest (ROI) analyses, template‐space WM ROIs from the JHU DTI atlas^19^ were warped into subject‐space, and mean DTI measures from each ROI for each time point were extracted.

T1‐weighted images were bias field‐corrected and segmented into GM, WM, and cerebrospinal fluid (CSF) using an adaptive maximum *a posteriori* (AMAP) approach^20^ in the Computational Anatomy Toolbox (CAT12, http://www.neuro.uni‐jena.de/cat/) in SPM12 (https://www.fil.ion.ucl.ac.uk/spm/). Prior to further processing, all segmentation maps were visually inspected; furthermore, quantitative CAT12 quality ratings (scored out of 100) did not differ between treatment groups (84.7 ± 1.3 for placebo vs 84.5 ± 1.8 for simvastatin; *t*‐test, *t* = 1.22, *P* = 0.22). Total relative volumes of GM and WM were calculated by dividing total GM and WM volumes by total intracranial volume (TIV; the total sum of GM, WM, and CSF volumes).

White matter hyperintensities (WMHs) were segmented using T1‐weighted and FLAIR images and the lesion growth algorithm^21^ (default kappa threshold of 0.3) as implemented in the LST toolbox version 2.0.15 (www.statisticalmodelling.de/lst.html) for SPM12. Lesion maps were all visually inspected prior to further analysis.

### Tract‐based spatial statistics (TBSS) processing

We performed voxel‐wise tract‐based spatial statistics (TBSS)^22^ in order to assess both baseline differences in WM microstructure between treatment groups, as well as the regional effects of simvastatin on longitudinal WM microstructure. To avoid bias from treatment or temporal effects when normalizing subject images to a common voxel space, we first used iterative groupwise non‐linear registration in ANTs to generate a within‐subject longitudinal average FA image for each subject using FA maps from all four time points. These within‐subject average FA images were then used as inputs to construct an unbiased population‐space template, which was subsequently non‐linearly registered to FSL’s “FMRIB58_FA” standard‐space image.^23^ Subject‐space DTI parameter maps were then aligned to standard‐space using the warp fields generated during the previous normalization steps (e.g., generating within‐subject average images, constructing the population‐space template, and registering to standard‐space). Standard‐space FA images were merged and averaged to create a mean FA image, which was skeletonized and thresholded at 0.3. Finally, each subject’s aligned FA, MD, AD, and RD parameter maps for all time points were projected onto this skeleton and used as inputs in voxel‐wise statistical analyses.

### Statistical analysis

All statistical analyses (except voxel‐wise analyses) were performed using R, version 3.6.1 (R Foundation for Statistical Computing). In order to assess the effect of simvastatin on longitudinal WM microstructure, mean DTI measures in global WM were expressed as percent change from baseline at 6, 12, and 18 months, and ANCOVA models were used to assess differences between simvastatin and placebo groups at each time point. Identical models tested treatment × sex and treatment × *APOE*‐ɛ4 genotype interactions. ANCOVA models were used to evaluate percent change in total relative GM, WM, and WMH lesion volume (all corrected for TIV) at each time point. All models included age, sex, and *APOE*‐ɛ4 genotype as covariates.

Voxel‐wise TBSS analyses were performed using skeletonized standard‐space DTI parameter maps in FSL’s *randomise* ^24^ (10,000 permutations) followed by threshold‐free cluster enhancement.^25^ Baseline differences between treatment groups were assessed using baseline DTI maps, while longitudinal analyses used maps representing percent change in DTI measures at 18 months (calculated as [18m image – baseline image]/baseline image × 100). Separate contrasts tested both simvastatin group > placebo group and simvastatin group < placebo group for baseline and longitudinal analyses for all DTI metrics, and all analyses included age, sex, and *APOE*‐ɛ4 genotype as covariates. Resulting statistical maps were family‐wise error (FWE)‐corrected at *P*_FWE_ < 0.05. To provide quantification of longitudinal TBSS results, significant voxels from the statistical maps were deprojected from the skeleton and warped back into each subject’s native imaging space. Mean values across all significant voxels were then extracted from the respective DTI parameter maps for each subject.

Follow up exploratory voxel‐based morphometry (VBM) analyses were performed to investigate regional effects of simvastatin on WM volume. Template‐space WM segmentation maps (generated by default during CAT12 processing) representing percent change in WM at 12 and 18 months were used in *randomise* (10,000 permutations with threshold‐free cluster enhancement), and models included age, sex, and *APOE*‐ɛ4 genotype as covariates.

Finally, we tested whether the effects of simvastatin on WM microstructure were mediated by change in serum cholesterol levels with the application of the counterfactual framework mediation analysis method^26^ using the mediation package (version 4.5.0) in R. Confidence intervals for the indirect (mediation), direct, and total effects were constructed using nonparametric bootstrapping with 10,000 bootstrapped samples. In these analyses, the mediator variable was percent change in serum total cholesterol at 18 months, and the outcome variable was mean percent change in DTI metrics at 18 months within the deprojected significant voxels (from the respective TBSS analyses for each DTI metric). A treatment×mediator interaction term was included in all models. Age, sex, and *APOE*‐ɛ4 genotype were included as covariates.

## Results

### Participant characteristics and changes in serum cholesterol levels

Simvastatin and placebo groups did not differ with respect to demographic, laboratory, or neuroimaging measures at study baseline (Table 2). At all follow‐up time points, the simvastatin‐treated participants had significantly lower serum LDL cholesterol and total cholesterol relative to the placebo‐treated participants (Table 3). By 18 months, LDL cholesterol had decreased by 42.5% (53 ± 23 mg/dL) in the simvastatin group compared to 5.5% (8 ± 23 mg/dL) in the placebo group, and total cholesterol had decreased by 25.1% (53 ± 25 mg/dL) in the simvastatin group compared to 3.3% (9 ± 31 mg/dL) in the placebo group.

**Table 2:** Participant demographic, laboratory characteristics, and neuroimaging measures at study baseline.

Characteristic	Placebo (*n* = 38)	Simvastatin (*n* = 35)	*P* Value
Age, mean (SD), y	55.7 (8.0)	56.7 (6.3)	0.57
Female sex, No. (%)	29 (76.3)	24 (68.6)	0.63
White/Caucasian race, No. (%)	37 (97.4)^a^	35 (100)	>0.99
*APOE*‐ɛ4 positive, No. (%)	14 (36.8)	14 (40.0)	0.97
ASCVD 10‐y risk score, median [IQR]	2.3 [1.2‐4.8]	2.8 [1.6‐6.7]	0.38
BMI, mean (SD)	28.0 (5.8)	28.0 (6.0)	0.98
Blood pressure			
Systolic, mean (SD), mm Hg	124 (18)	125 (17)	0.89
Diastolic, mean (SD), mm Hg	73 (11)	73 (11)	0.73
Antihypertensive medication use, No. (%)	7 (18.4)	6 (17.1)	>0.99
Current smoker, No. (%)	1 (2.6)	3 (8.6)	0.55
Alcohol use in last month, No. (%)	30 (78.9)	30 (85.7)	0.65
Serum lipid profile (fasting)			
Total cholesterol, mean (SD), mg/dL	209 (35)	205 (37)	0.56
Triglycerides, mean (SD), mg/dL	103 (41)	112 (58)	0.45
HDL‐C, mean (SD), mg/dL	64 (17)	62 (21)	0.75
LDL‐C, mean (SD), mg/dL	125 (29)	120 (30)	0.45
Neuroimaging measures			
DTI metrics (global white matter)			
Fractional anisotropy (FA), mean (SD)	0.424 (0.014)	0.428 (0.013)	0.15
Mean diffusivity (MD), mean (SD), 10^−3^ mm^3^/s	0.785 (0.024)	0.784 (0.027)	0.83
Radial diffusivity (RD), mean (SD), 10^−3^ mm^3^/s	0.591 (0.024)	0.587 (0027)	0.54
Axial diffusivity (AD), mean (SD), 10^−3^ mm^3^/s	1.175 (0.028)	1.178 (0.030)	0.61
Total GM volume, mean (SD), mL	627 (56)	633 (58)	0.67
Total WM volume, mean (SD), mL	508 (60)	508 (61)	0.98
Total intracranial volume (TIV), mean (SD), mL	1436 (134)	1446 (130)	0.75
Total WMH lesion volume, median [IQR], mL	0.72 [0.19‐1.47]	0.45 [0.17‐1.69]	0.81

Abbreviations: *APOE*, apolipoprotein E; ASCVD, atherosclerotic cardiovascular disease; BMI, body mass index; DTI, diffusion tensor imaging; GM, gray matter; HDL‐C, high‐density lipoprotein cholesterol; LDL‐C, low‐density lipoprotein cholesterol; WM, white matter; WMH, whitematter hyperintensity.

^a^ One American Indian/Native American participant in placebo group.

**Table 3:** Fasting serum lipid profiles for simvastatin and placebo groups at 6, 12, and 18 months.

Serum lipid profile (fasting)	6 months			12 months			18 months		
	Placebo (*n* = 38)	Simvastatin (*n* = 35)	*P* Value	Placebo (*n* = 38)	Simvastatin (*n* = 35)	*P* Value	Placebo (n = 38)	Simvastatin (n = 35)	*P* Value
Total cholesterol, mean (SD), mg/dL	203 (29)	142 (25)	**<0.001**	204 (32)	148 (28)	**<0.001**	200 (31)	152 (26)	**<0.001**
Triglycerides, mean (SD), mg/dL	109 (64)	88 (45)	0.11	102 (44)	96 (50)	0.60	113 (83)	91 (48)	0.17
HDL‐C, mean (SD), mg/dL	64 (19)	63 (19)	0.79	65 (19)	66 (22)	0.97	64 (22)	66 (20)	0.58
LDL‐C, mean (SD), mg/dL	117 (26)	62 (21)	**<0.001**	118 (29)	64 (19)	**<0.001**	117 (28)	67 (18)	**<0.001**

Abbreviations: HDL‐C, high‐density lipoprotein cholesterol; LDL‐C, low‐density lipoprotein cholesterol. Bold text represents significant *P* value < 0.05.

### Effect of simvastatin on WM microstructure

**Figure 2:**
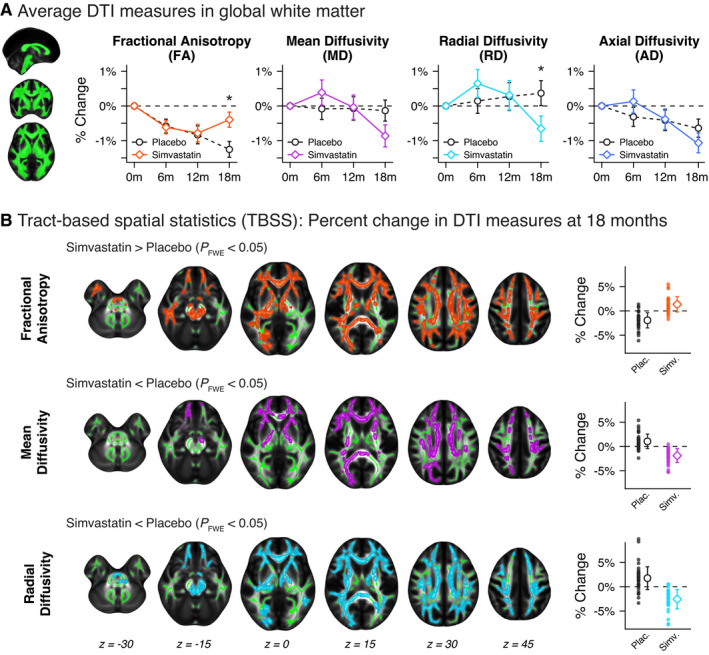
(A) Longitudinal trajectories of mean diffusion tensor imaging (DTI) metrics within global white matter (WM) for placebo and simvastatin groups over 18 months. At 18 months, the percent change in fractional anisotropy (FA) and radial diffusivity (RD) in global WM was significantly different between treatment groups. *P < 0.05. (B) Tract‐based spatial statistics (TBSS) results showing voxels in WM with significantly different percent change in DTI measures at 18 months for FA, mean diffusivity (MD), and RD (10,000 permutations, familywise error‐corrected *P* < 0.05). There were no significant voxels for axial diffusivity (AD). Dot plots to the right of axial slices show the mean percent change (± standard deviation) within significant voxels for placebo and simvastatin groups.

We first tested the effects of simvastatin treatment on global WM microstructure. At 18 months, the percent change in FA and RD in global WM was significantly different between treatment groups (Fig. 2A; Supplemental Table S2). Specifically, change in FA from baseline at 18 months was −1.25 ± 0.23% in the placebo group compared to −0.40 ± 0.22% in the simvastatin group (adjusted difference in group means 0.88%, 95% CI 0.27 to 1.50, *P* = 0.005). Additionally, change in RD from baseline at 18 months was 0.37 ± 0.36% in the placebo group compared to −0.66 ± 0.36% in the simvastatin group (adjusted difference in group means −1.10%, 95% CI −2.13 to −0.06, *P* = 0.039). There were no significant differences between treatment groups for MD and AD in global WM, and no significant treatment × sex or treatment×*APOE*‐ɛ4 genotype interactions for any DTI metrics.

We next used voxel‐wise TBSS analyses in order to further assess the effects of simvastatin on regional WM microstructure. There were significant differences between treatment groups for 18‐month percent change in FA, MD, and RD throughout extensive regions of the WM skeleton, with more widespread effects observed for FA and RD than MD (Fig. 2B). Significant voxel clusters were located in areas corresponding to major WM tracts (corpus callosum, corticospinal tract, anterior thalamic radiation, inferior longitudinal fasciculus, superior longitudinal fasciculus, inferior fronto‐occipital fasciculus) as well as brainstem WM regions (cerebellar peduncle). Within significant clusters, FA change from baseline was −1.9 ± 1.6% in the placebo group compared to 1.4 ± 1.6% in the simvastatin group; MD and RD change from baseline was 1.1 ± 1.5% and 1.8 ± 2.3%, respectively, in the placebo group compared to −1.9 ± 1.4% and −2.6 ± 2.0%, respectively, in the simvastatin group. Notably, there were no significant voxels where FA percent change was lower, or where MD or RD percent change was higher, in the simvastatin group compared to the placebo group after 18 months. Additionally, there were no significant voxels where AD percent change was different between treatment groups. An ROI analyses using predefined WM ROIs from the JHU tractography atlas showed similar results (Supplemental Fig. S1). Out of eleven ROIs, FA was significantly altered in nine regions, MD was significantly altered in four regions, and RD was significantly altered in seven regions. The simvastatin group showed the greatest differences in corpus callosum (forceps major and minor) and inferior fronto‐occipital fasciculus.

### Effect of simvastatin on GM, WM, and WMH volume

**Figure 3:**
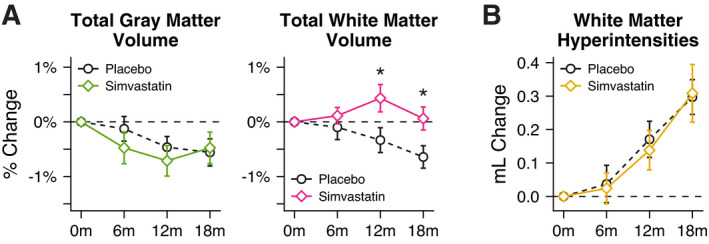
Longitudinal trajectories of (A) gray matter (GM) volume, white matter (WM) volume, and (B) white matter hyperintensity (WMH) lesion volume for placebo and simvastatin groups over 18 months. The simvastatin group showed significantly different percent change in total WM volume at 12 and 18 months compared to the placebo group. There were no significant differences in GM or WMH volume changes between placebo and simvastatin groups. * *P* < 0.05.

While there were no significant effects of treatment on longitudinal total GM volume, the simvastatin group showed significantly different percent change in total WM volume at 12 and 18 months compared to the placebo group (Fig. 3A). At 12 months, change in WM volume from baseline was −0.33 ± 0.23% in the placebo group compared to 0.43 ± 0.25% in the simvastatin group (adjusted difference in group means 0.77%, 95% CI 0.10 to 1.43, *P* = 0.026). At 18 months, change in WM volume from baseline was −0.64 ± 0.20% in the placebo group compared to 0.06 ± 0.21% in the simvastatin group (adjusted difference in group means 0.72%, 95% CI 0.13 to 1.32, *P* = 0.018). Exploratory VBM analyses showed that these effects were distributed throughout WM, with strongest effects occurring in bilateral anterior temporal and superior frontal WM regions (Supplemental Fig. S2). There were no significant treatment effects on longitudinal WMH lesion volume (Fig. 3B).

### Mediation of change in WM microstructure by change in serum total cholesterol

**Figure 4:**
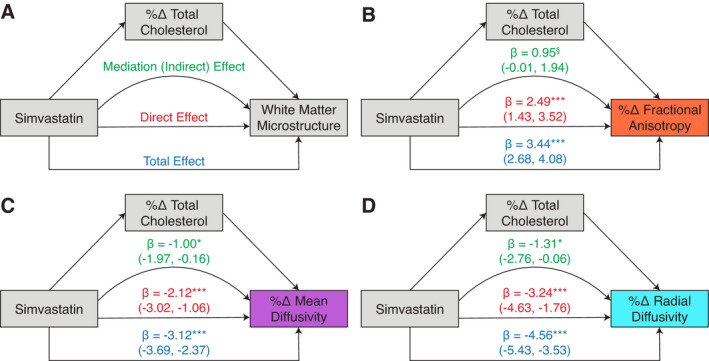
Models and results for mediation analyses for change in total cholesterol mediating the effect of simvastatin on change in white matter microstructure. (A) Conceptual model of mediation pathway and model parameters. For each model, the mediator variable was percent change in serum total cholesterol at 18 months, and the white matter microstructure outcome was mean percent change in DTI metrics within significant voxels (from the TBSS analyses) at 18 months. For all three DTI metrics (B‐D), the effect of simvastatin on change in white matter microstructure was partially mediated by the percent change in total cholesterol. Beta‐coefficients are shown for the mediation, direct, and total effects, along with bootstrapped 95% confidence intervals (10,000 iterations). ^§^ *P* = 0.06; * *P* < 0.05; *** *P* < 0.001.

We found that the effect of simvastatin on change in WM microstructure was partially mediated by the percent change in serum total cholesterol (Fig. 4). Change in serum total cholesterol mediated 28%, 32%, and 29% of the total effect for FA, MD, and RD, respectively. Visualizing this relationship showed that individuals with the greatest change in total cholesterol levels had the generally had the greatest change in DTI measures (Supplemental Fig. S3).

## Discussion

In this analysis of longitudinal neuroimaging data acquired during a randomized controlled trial, simvastatin treatment in healthy, statin‐naïve, cognitively unimpaired adults resulted in significantly altered WM microstructure and volume over 18 months compared to placebo treatment. Specifically, while the placebo group showed longitudinal changes consistent with gradual age‐related reductions in WM microstructure (decreased FA and AD, increased RD),^27^, ^28^, ^29^ the simvastatin group showed distinctly altered trajectories, such that by 18 months, decline in FA was significantly attenuated and RD was decreased relative to baseline. Additionally, longitudinal TBSS analyses demonstrated that these effects were widely distributed throughout cerebral and brainstem WM. Notably, within significant voxels, placebo, and simvastatin groups showed WM microstructural changes in opposing directions, suggesting an effect of simvastatin that is opposite to apparent age‐related changes. Finally, while the placebo group showed gradual decline in WM volume, the simvastatin group showed virtually no change from baseline in WM volume at 18 months. Taken together, these results suggest that simvastatin treatment resulted in preserved WM microstructure and volume over 18 months compared to placebo treatment.

Given its sensitivity to restricted diffusion of water molecules, DTI provides insight in WM architecture. Within WM, RD (the magnitude of diffusivity perpendicular to the principle direction of hindered diffusion) and AD (the magnitude of diffusivity along the principle direction of hindered diffusion) are commonly regarded as surrogate measures of myelin and axonal integrity, respectively.^30^, ^31^, ^32^, ^33^ In the current study, simvastatin treatment resulted in significant changes in RD, but not AD, suggesting an effect on myelin, but not axonal integrity. Interestingly, the simvastatin group showed decreased RD from baseline at 18 months, potentially indicating increased myelin content as a result of statin treatment. However, DTI metrics are indirect measures of tissue microstructure, and histopathological correlation is required to confirm these changes.

Results from prior observational studies of the effects of statins on WM microstructure have been mixed. In a study of community‐dwelling elderly adults, statin‐exposure was associated with higher FA in global WM, but only in individuals in the lowest tertile of cognitive performance.^9^ Alternatively, in a large population‐based study of >1100 older adults, long‐term (>5 yrs) statin exposure was associated with lower FA in the genu of the corpus callosum, although this finding was driven by increased cardiovascular disease risk factors in statin‐exposed individuals.^10^ It is important to note that statin type, dose, and duration of use differed within the statin‐exposed individuals in both of these studies, and it is unknown whether WM microstructure alterations existed prior to initiation of statin treatment. In the current study, the use of data collected during a randomized control trial in statin‐naïve adults provides strong evidence that simvastatin impacts WM microstructure over time, even in cognitively unimpaired individuals with low cardiovascular disease risk.

Statins may affect WM through both peripheral and central pathways.^34^ One potential peripheral pathway is through lowering circulating cholesterol levels, and previous studies have shown that both higher LDL cholesterol and lower HDL cholesterol levels are associated with lower WM microstructural integrity in cognitively unimpaired older adults.^35^, ^36^, ^37^ In the current study, we tested this peripheral pathway using mediation analysis and found that approximately 30% of the effect of simvastatin on WM microstructure was mediated by changes in serum total cholesterol levels. Although the exact mechanisms by which reducing peripheral cholesterol may affect brain WM are uncertain, lower circulating cholesterol may reduce atherosclerosis and improve cerebrovascular health, even in individuals with generally low vascular disease risk. Additionally, reduction in serum cholesterol may be a surrogate measure for other peripheral effects of statins, including reducing endothelial dysfunction and vascular inflammation.^38^ In the present study, it is worth noting that simvastatin treatment did not appear to affect WMH lesion volume, a commonly used neuroimaging marker of small vessel cerebrovascular disease.^39^ However, the pathophysiology of WMH burden is multifactorial and may be more attributed to hypertension,^40^, ^41^ which was unaffected by simvastatin. Additionally, peripheral mechanisms including improved endothelial dysfunction and reduced vascular inflammation would likely not be detected with WMH lesion volume, and future studies directly measuring peripheral vascular markers and WM health are needed.

While mediation analyses suggest that lowering peripheral cholesterol levels contributed to a portion of the effect of simvastatin on preserving WM integrity, they also indicate that a majority of the effect of simvastatin on WM microstructure occurred through other pathways, including central pathways. Simvastatin is a lipophilic statin capable of crossing the BBB and may affect WM by directly affecting cholesterol homeostasis in the brain. Previous clinical studies have shown that statin treatment not only generally reduces brain cholesterol synthesis, but also decreases brain cholesterol efflux,^42^, ^43^, ^44^, ^45^ suggesting compensatory mechanisms to maintain cholesterol levels in the brain. Notably, a study evaluating the effects of long‐term statin treatment (>12 months) observed initial reduction in brain cholesterol synthesis and metabolites, followed by a return to baseline and overshoot at 2 years.^45^ Intriguingly, this pattern of compensatory cholesterol metabolism resembles the trajectories of DTI metrics observed for the simvastatin group in the current study. This was particularly the case for RD, which showed an initial increase at 6 months followed by eventual decrease at 18 months. Finally, in addition to directly affecting brain cholesterol homeostasis, statins also have pleotropic effects in the brain.^7^ By inhibiting production of brain isoprenoids (a downstream intermediate in the cholesterol synthesis pathway), statins can reduce neuroinflammation and oxidative stress,^46^, ^47^ which may have beneficial effects on WM integrity. Overall, simvastatin treatment likely leads to preservation of WM through both peripheral and central effects, in which improvement in cerebrovascular health may be augmented by upregulating brain cholesterol recycling or reducing neuroinflammation.

While a major strength of the current study is the use of longitudinal data collected during a randomized controlled trial, the treatment groups were relatively small and were racially homogenous. Additionally, all participants had a parental family history of Alzheimer’s disease dementia. Thus, it remains to be determined whether the effects of simvastatin on WM generalize to larger samples that are more representative of the general population. However, in the current study, there was no evidence of interaction between treatment and *APOE*‐ɛ4 genotype, suggesting that the effects of simvastatin are independent of Alzheimer’s disease genetic risk. Additionally, this study included a relatively large age range of participants (40–72 years old), and the small sample size did not allow us to determine whether the effects of simvastatin on WM are more pronounced in certain ages. Finally, future studies extending longer than 18 months are needed in order to determine the longevity and persistence of the effects of simvastatin on WM integrity.

## Conclusions

Statins are prescribed for millions of adults, yet whether these medications affect cholesterol‐rich WM regions in the brain is largely unknown. Here, we show that simvastatin, a lipophilic statin capable of crossing the BBB, preserves WM microstructure and volume in healthy, cognitively unimpaired middle‐aged adults. While statins have been proposed as therapeutic agents for neurodegenerative conditions, including Alzheimer’s disease, clinical trials have largely been negative,^48^, ^49^ potentially due to inadequate duration of therapy or intervention occurring well after development of cognitive impairment. Future studies are needed to replicate these findings and determine whether the effects of simvastatin on WM structure translate into delaying or preventing cognitive changes.

## Author Contributions

NMV, JFVH, YM, CAV, RJC, KLK, LEJ, BPA, SA, SCJ, BBB, and CMC were involved in conception and design of the study. NMV, JFVH, YM, CAV, NA, RJC, KLK, LEJ, and BPA assisted in acquisition and analysis of data. NMV, JFVH, BBB, and CMC were involved in drafting the manuscript.

## Conflicts of Interest

Nothing to report.

## Supporting information


**Supplemental File S1.** Supplemental material including tables and figures.
