# Supplementary material for: Effects of simvastatin on white matter integrity in healthy middle‐aged adults
Source: Ann Clin Transl Neurol. 2021 Jul 18;8(8):1656–67. doi: 10.1002/acn3.51421 (PMC8351379; doi:10.1002/acn3.51421)

Supplemental Material

Effects of simvastatin on white matter integrity in healthy middle-aged adults: a secondary analysis of a randomized controlled trial

Nicholas M Vogt, PhD; Jack F V Hunt, PhD; Yue Ma, PhD; Carol A Van Hulle, PhD; Nagesh Adluru, PhD; Richard J Chappell, PhD; Karen K Lazar, MS; Laura E Jacobson, MPH; Benjamin P Austin, PhD; Sanjay Asthana, MD; Sterling C Johnson, PhD; Barbara B Bendlin, PhD; Cynthia M Carlsson, MD, MS*

Supplemental Table 1. Baseline participant characteristics for full study population (n=88)

	Participants, No. (%)		
**Characteristic**	**Placebo (n=44)**	**Simvastatin (n=44)**	***P* Value**
Age, mean (SD), y	54.8 (7.8)	56.2 (6.5)	0.36
Female sex	32 (74.4)	31 (70.5)	>0.99
White/Caucasian race	43 (97.7)[a]	44 (100)	>0.99
APOE-ε4 positive	17 (38.6)	17 (38.6)	>0.99
ASCVD 10-y risk score, median [IQR]	2.2 [1.0-3.8]	2.9 [1.4-4.4]	0.24
BMI, mean (SD)	27.6 (5.6)	27.2 (5.6)	0.75
Blood pressure			
Systolic, mean (SD), mm Hg	124 (17)	125 (16)	0.69
Diastolic, mean (SD), mm Hg	73 (11)	72 (9.9)	0.85
Serum lipid profile (fasting)			
Total cholesterol, mean (SD), mg/dL	207 (34)	207 (36)	0.94
Triglycerides, mean (SD), mg/dL	102 (40)	112 (58)	0.32
HDL-C, mean (SD), mg/dL	63 (17)	63 (21)	0.82
LDL-C, mean (SD), mg/dL	123 (28)	122 (28)	0.93

Abbreviations: *APOE*, apolipoprotein E; ASCVD, atherosclerotic cardiovascular disease; BMI, body mass index; HDL-C, high-density lipoprotein cholesterol; LDL-C, low-density lipoprotein cholesterol

[a]One American Indian/Native American participant in placebo group

Supplemental Table 2. Summary of ANCOVA models for effect of simvastatin treatment on diffusion tensor imaging measures

	**6 months** β coefficient (SE)				**12 months** β coefficient (SE)				**18 months** β coefficient (SE)			
**Variable**	**FA**	**MD**	**RD**	**AD**	**FA**	**MD**	**RD**	**AD**	**FA**	**MD**	**RD**	**AD**
Treatment – simvastatin	-0.02 (0.28)	0.44 (0.48)	0.47 (0.54)	0.42 (0.43)	0.07 (0.37)	0.0005 (0.50)	-0.002 (0.59)	0.008 (0.43)	**0.88** (0.31)**	-0.79 (0.44)	**-1.10* (0.52)**	-0.48 (0.39)
Age	-0.03 (0.02)	0.06 (0.03)	0.07 (0.04)	0.05 (0.03)	0.002 (0.03)	0.01 (0.04)	0.01 (0.04)	0.01 (0.03)	0.02 (0.02)	0.05 (0.03)	0.04 (0.04)	0.05 (0.03)
Sex – Male	0.24 (0.32)	-0.43 (0.54)	-0.50 (0.61)	-0.35 (0.49)	-0.19 (0.42)	-0.007 (0.56)	0.10 (0.67)	-0.10 (0.48)	**-0.81* (0.35)**	0.24 (0.50)	0.55 (0.59)	-0.07 (0.44)
APOE-ε4 – positive	-0.07 (0.29)	0.17 (0.50)	0.19 (0.56)	0.15 (0.45)	-0.18 (0.39)	0.66 (0.52)	0.72 (0.62)	0.59 (0.45)	**0.67* (0.32)**	0.09 (0.46)	-0.19 (0.54)	0.37 (0.40)

Abbreviations: *APOE*, apolipoprotein E; FA, fractional anisotropy; MD, mean diffusivity; RD, radial diffusivity; AD, axial diffusivity; SE, standard error

* $P < 0.05$, ** $P < 0.01$

Supplemental Figure 1. Region of interest (ROI) analysis showing 18-month percent change in WM DTI measures. Points represent mean (± SEM) for each ROI (averaged bilaterally, except for forceps major and forceps minor). **P* < 0.05 (uncorrected); ANCOVA models controlling for age, sex, *APOE*-ε4 genotype. Abbreviations: ATR, Anterior thalamic radiation; CST, corticospinal tract; CCG, cingulum cingulate gyrus; CHB, cingulum hippocampal bundle; IFOF, inferior fronto-occipital fasciculus; ILF, inferior longitudinal fasciculus; SLF, superior longitudinal fasciculus; UF, uncinate fasciculus; SLFT, superior longitudinal fasciculus (temporal part); Fmaj, forceps major; Fmin, forceps minor

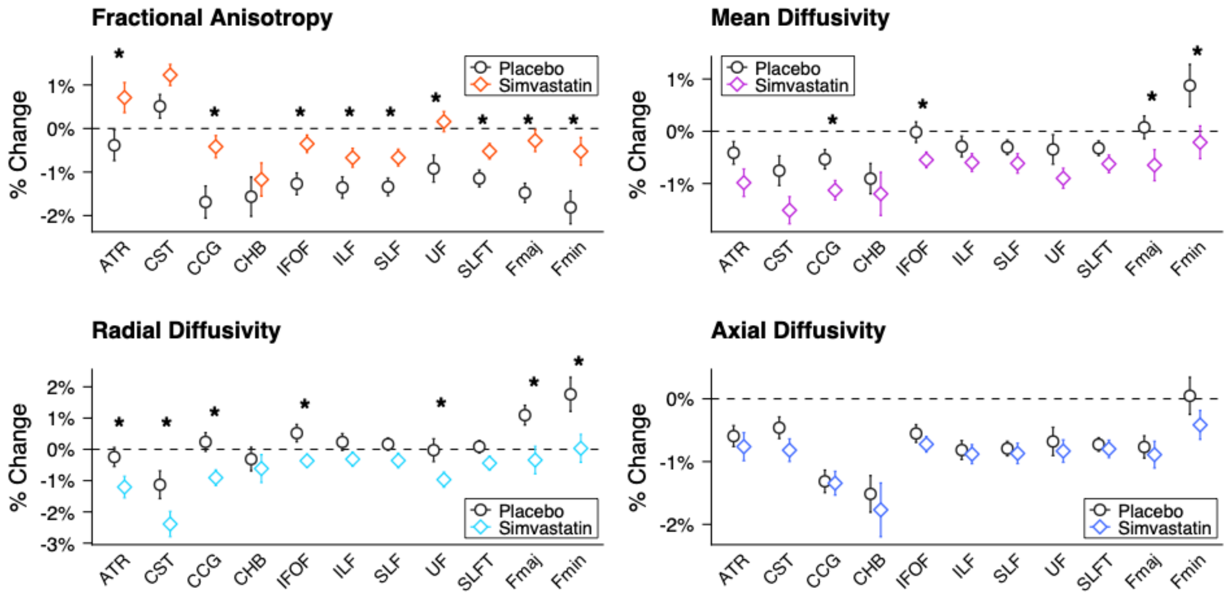

Supplemental Figure 2. Exploratory voxel-based morphometry (VBM) analyses of 12- and 18-month percent change in WM volume (Simvastatin > Placebo, P_{uncorr} < 0.01, n =10,000 permutations followed by threshold-free cluster enhancement).

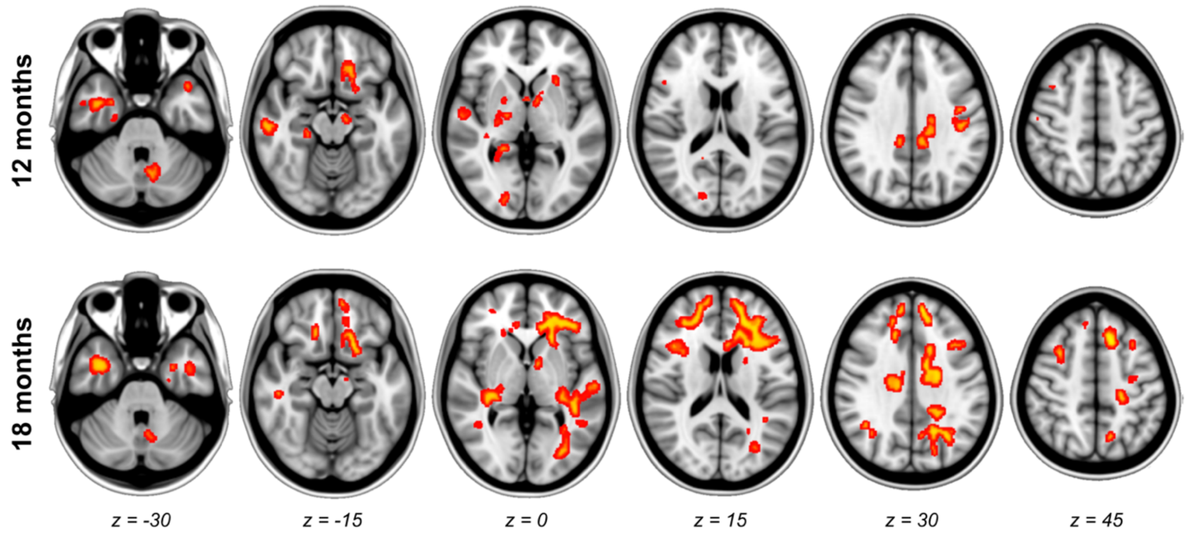

Supplemental Figure 3. 18-month percent change in total cholesterol vs. 18-month change in DTI neuroimaging measures. Abbreviations: FA, fractional anisotropy; MD, mean diffusivity; RD, radial diffusivity

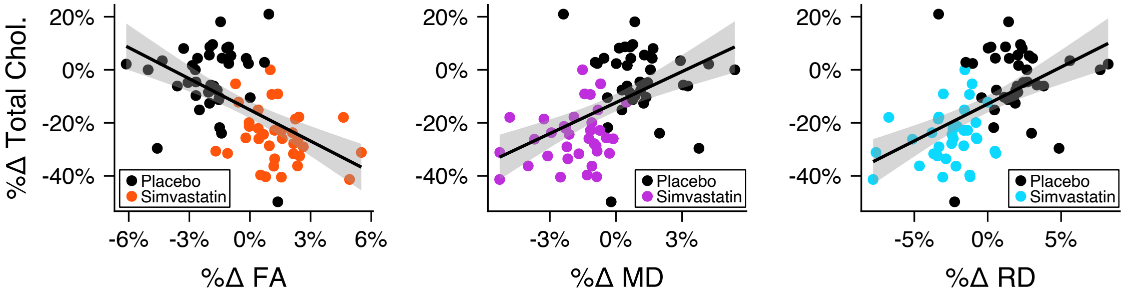

Supplement: Supplementary file 1 — Supplemental File S1. Supplemental material including tables and figures. [file ACN3-8-1656-s001.pdf]
